# Boerhaave’s Syndrome: A Case Report

**DOI:** 10.7759/cureus.23836

**Published:** 2022-04-05

**Authors:** Tiago Ceriz, Andreia Diegues, João Lagarteira, Rui Terras Alexandre, Andrés Carrascal

**Affiliations:** 1 Internal Medicine Department, Unidade Local de Saúde do Nordeste, Bragança, PRT; 2 Intensive Medicine and Emergency Department, Unidade Local de Saúde do Nordeste, Bragança, PRT

**Keywords:** thoracic drainage, pleural effusion, hydropneumothorax, esophagus perforation, boerhaave’s syndrome

## Abstract

Boerhaave’s syndrome is a rare spontaneous perforation of the esophagus with high mortality. The diagnosis is difficult because it has no specific symptom. It requires urgent intervention. We report the case of a 63-year-old male admitted to the emergency department with respiratory distress. Chest computed tomography (CT) revealed large, bilateral, and tension hydropneumothorax, stomach distention, and aerobilia. Thoracic drainage was performed immediately. Control CT revealed esophagus perforation in the middle third of the esophagus with extravasation of the contrast product from the esophagus to the pleura. Urgent surgery was performed, and the patient was admitted to the intensive care unit (ICU) with septic shock. Early diagnosis and management with a damage control approach including thoracic drainage and surgery are essential to improve prognosis and reduce mortality.

## Introduction

Boerhaave’s syndrome is a rare, life-threatening, spontaneous perforation of the esophagus, requiring urgent diagnosis and treatment. It was described for the first time by Herman Boerhaave in 1729. It is associated with high morbidity and mortality. The majority of Boerhaave’s syndrome occurs in individuals with a normal underlying esophagus; in some cases, eosinophilic esophagitis, medication-induced esophagitis, Barrett’s esophagus, or infectious ulcers can be observed. This results from a sudden intraesophageal pressure increase combined with negative intrathoracic pressure [1]. The rupture leads to mediastinal inflammation, with pleural rupture subsequently. Clinical manifestation depends on the location of the perforation. Normally, excruciating retrosternal chest pain due to an intrathoracic esophageal perforation is the typical sign, with frequently severe retching and vomiting preceding the onset of pain. Pleural effusion may also be detected within hours of the perforation, as well as subcutaneous emphysema. As with intrathoracic perforation, sepsis may rapidly develop too. The diagnosis is challenging because of its rarity and nonspecific symptoms; the majority of cases are diagnosed incidentally. Imaging examinations are crucial. Management requires a multidisciplinary team [2-7].

## Case presentation

A 63-year-old foreign male truck driver was admitted to the emergency room for respiratory distress. In his belongings, we found a box of trimethoprim-sulfamethoxazole; collecting more information was difficult due to the language barrier. Physical examination showed respiratory distress, absent pulmonary sounds, hypoxia (74%), hemodynamic instability with a blood pressure of 95/45 mmHg, tachycardia (heart rate: 106 bpm), and signs of peritoneal irritation, especially in the epigastric region. Arterial blood gas analysis showed mixed acidosis, severe hypoxia, and hyperlacticemia (Table 1).

**Table 1:** Arterial blood gas analysis (21% FiO2) at admission showing mixed acidosis, severe hypoxia, and hyperlacticemia

Parameter	Result	Normal range
pH, mmHg	7.10	7.350-7.450
pCO2, mmHg	55.3	35-45
pO2, mmHg	35.5	75-100
HCO3-, mmol/L	18	22-26
SO2, %	68	94-100
Lactate, mmol/L	6.4	0.5-2

**Figure 1:**
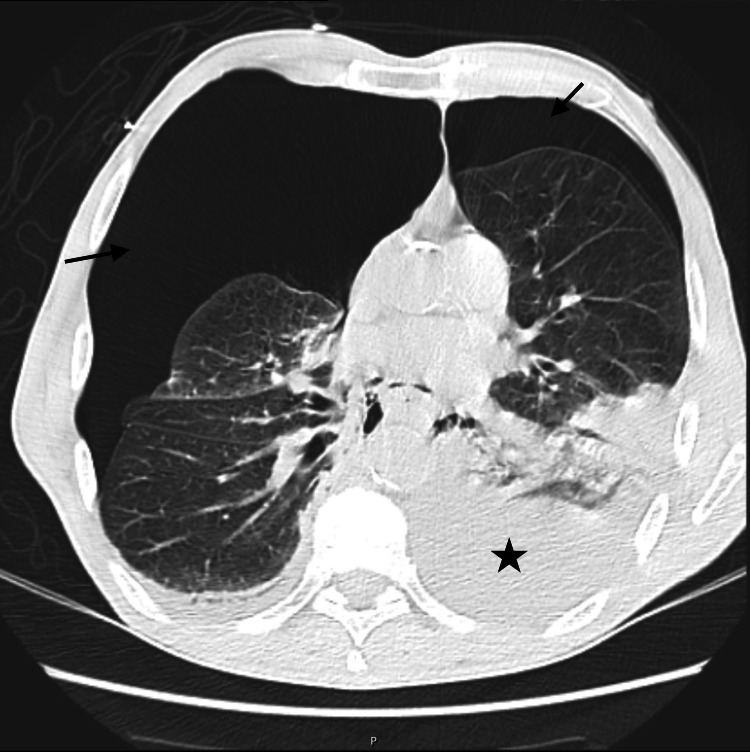
First CT scan Chest computed tomography (CT) revealed large, bilateral tension hydrothorax (star) and pneumothorax (arrow). Increased pleural effusion can be observed in the left hemithorax and atelectasis in the right medial lobe and both inferior lobes with reduction of the total lung volume predominantly on the right side.

Chest computed tomography (CT) revealed large, bilateral, and tension hydropneumothorax, stomach distention, and aerobilia (Figure 1).

**Figure 2:**
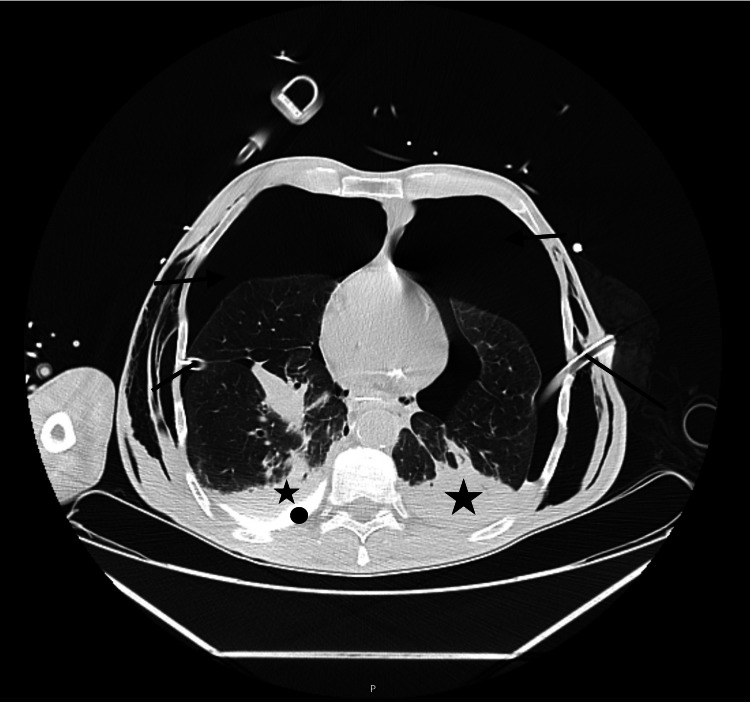
Second CT scan after the thoracic drainage technique Extensive high-volume bilateral hydrothorax (star) and pneumothorax (arrow) associated with pleural effusion (circle) are shown, as well as the bilateral thoracic drainage tubes in the pleural cavity (line).

**Figure 3:**
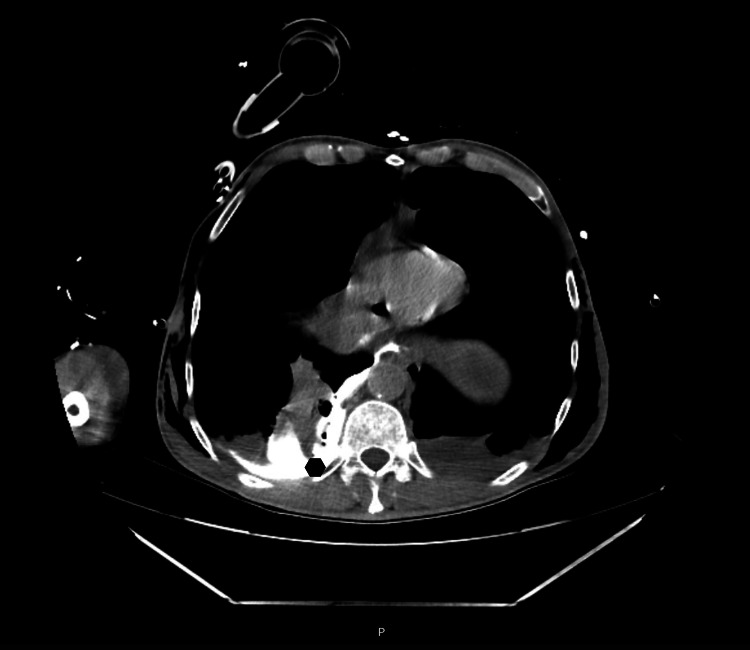
Second CT scan after the thoracic drainage technique On the right side, contrast extravasation from the esophagus in the pleural cavity (lozenge) is shown, indicating a probable lesion of the middle third of the esophagus.

**Figure 4:**
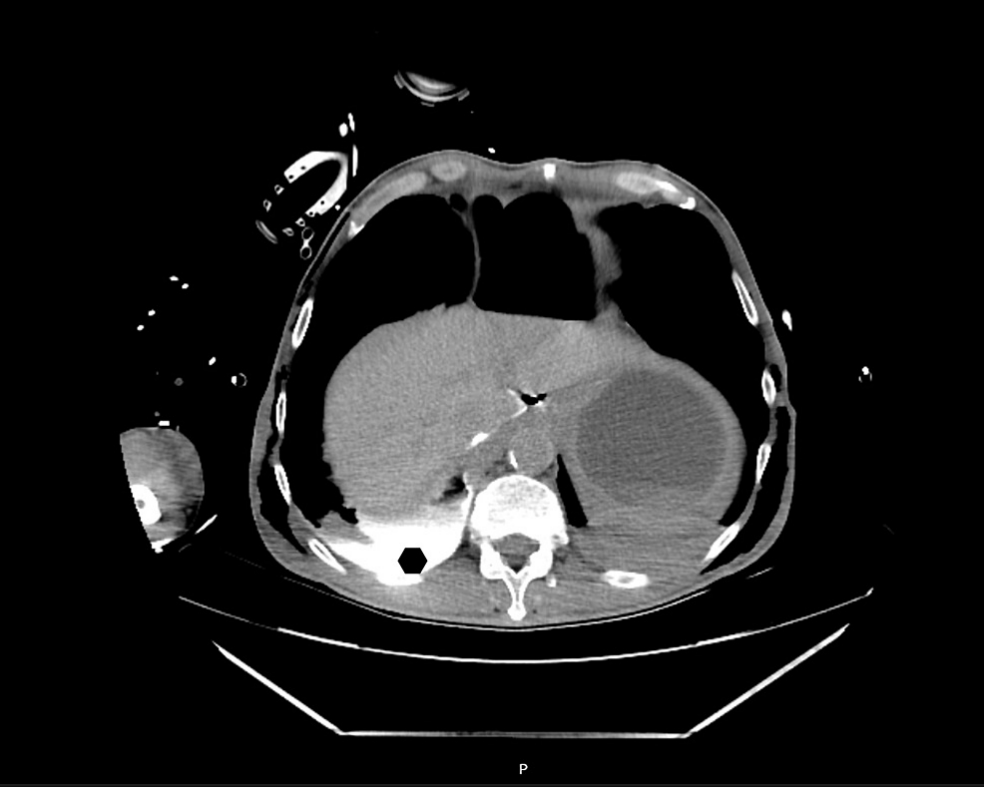
Second CT scan after the thoracic drainage technique Contrast marker on the pleural cavity clearly seen on the posterior right hemithorax (lozenge).

Thoracic chest drainage was performed immediately; bilateral chest tubes were inserted with enteric content drainage. The patient was sedated, and invasive mechanical ventilation was needed. The initial approach with fluid resuscitation and antibiotic was started. Blood tests have no relevant alterations. A CT was repeated showing extravasation of contrast product from the esophagus to the pleura due to injury in the middle third of the esophagus, pneumoperitoneum, and extensive subcutaneous emphysema; Boerhaave’s syndrome was suspected (Figures 2, 3, 4).

During surgery, duodenal stenosis was observed causing gastric distension, probably due to a fibrous ulcer, with intra-abdominal fluid, esophagus perforation, and pleural rupture at the level of the esophageal pillars. The surgical technique included esophageal and duodenal exclusion, antrectomy, gastroduodenal anastomosis, and jejunostomy for feeding. Postoperatively, the patient was admitted to the intensive care unit (ICU) with septic shock. The postoperative course was complicated with chemical peritonitis requiring new surgical intervention. The patient’s hospital stay was complicated with thrombosis of the internal jugular vein, delirium, and myopathy of the critical patient, as well as ventilatory weaning conditioning. After four months of evolution, he improved gradually and was discharged home.

## Discussion

As explained before, Boerhaave’s syndrome - spontaneous rupture - is uncommon, representing 15% of all esophageal ruptures. It is more frequent in men in the sixth and seventh decades. The rupture can occur in the cervical or intra-abdominal esophagus and most frequently occurs in the lower third of the esophagus and in the left lateral position, due to anatomic weakness in this area [8]. The clinical presentation is unspecific as described previously, so the diagnosis requires a high index of suspicion. The most common symptoms include emesis or retching, chest pain, and subcutaneous emphysema. In our case, we see respiratory distress with no pulmonary sounds on auscultation and epigastric tenderness. The diagnosis was made after imaging on chest CT. Laboratory findings are not specific as described [9]. The major differential diagnoses include spontaneous pneumothorax, gastric ulcer or bowel perforation with free air in the abdomen, diaphragmatic rupture, and pneumonia.

Esophageal rupture may lead to various serious complications, such as mediastinitis and peritonitis developing into sepsis, resulting in multiple organ failure syndrome, motivating ICU admission [10]. Therefore, it has a high mortality rate, ranging between 20% and 40%.

The appropriate method for the management of esophageal perforation depends on many factors, such as severity, time since perforation, location, age, and patient status upon presentation. Treatment could be conservative (antibiotics with percutaneous drainage of abscesses or collections) or surgical; there is no consensus. However, most series show good results with surgical repair, and it has been the definitive treatment. There have been few reports describing laparoscopic management of this entity, and there is new endoscopic management. The last one is well established at present and usually considered for patients with high-risk surgical intervention and those with a minimum leak. Boerhaave’s syndrome is managed on a case-by-case approach [11-16].

## Conclusions

Boerhaave’s syndrome still represents a diagnostic and therapeutic challenge. It should be contemplated in all situations with a combination of gastrointestinal symptoms, especially epigastric pain and pulmonary signs. An early diagnosis and management with damage control treatment including thoracic drainage and surgery are crucial. However, it is a high-mortality syndrome, with difficult management and without a standard treatment option, associated with a management rate of complications. This case is one of the rare occurrences of Boerhaave’s syndrome as rupture most commonly occurs in the left posterolateral wall of the distal third of the esophagus with extension into the left pleural cavity.
